# Accelerating Evidence Synthesis in Observational Studies: Development of a Living Natural Language Processing–Assisted Intelligent Systematic Literature Review System

**DOI:** 10.2196/54653

**Published:** 2024-10-23

**Authors:** Frank J Manion, Jingcheng Du, Dong Wang, Long He, Bin Lin, Jingqi Wang, Siwei Wang, David Eckels, Jan Cervenka, Peter C Fiduccia, Nicole Cossrow, Lixia Yao

**Affiliations:** 1 IMO Health, 9600 W Bryn Mawr Ave # 100, Rosemont, IL, 60018, United States; 2 Merck & Co, Inc, 126 East Lincoln Ave, Rahway, NJ, United States, 1 619-643-2693

**Keywords:** machine learning, deep learning, natural language processing, systematic literature review, artificial intelligence, software development, data extraction, epidemiology

## Abstract

**Background:**

Systematic literature review (SLR), a robust method to identify and summarize evidence from published sources, is considered to be a complex, time-consuming, labor-intensive, and expensive task.

**Objective:**

This study aimed to present a solution based on natural language processing (NLP) that accelerates and streamlines the SLR process for observational studies using real-world data.

**Methods:**

We followed an agile software development and iterative software engineering methodology to build a customized intelligent end-to-end living NLP-assisted solution for observational SLR tasks. Multiple machine learning–based NLP algorithms were adopted to automate article screening and data element extraction processes. The NLP prediction results can be further reviewed and verified by domain experts, following the human-in-the-loop design. The system integrates explainable articificial intelligence to provide evidence for NLP algorithms and add transparency to extracted literature data elements. The system was developed based on 3 existing SLR projects of observational studies, including the epidemiology studies of human papillomavirus–associated diseases, the disease burden of pneumococcal diseases, and cost-effectiveness studies on pneumococcal vaccines.

**Results:**

Our Intelligent SLR Platform covers major SLR steps, including study protocol setting, literature retrieval, abstract screening, full-text screening, data element extraction from full-text articles, results summary, and data visualization. The NLP algorithms achieved accuracy scores of 0.86-0.90 on article screening tasks (framed as text classification tasks) and macroaverage F1 scores of 0.57-0.89 on data element extraction tasks (framed as named entity recognition tasks).

**Conclusions:**

Cutting-edge NLP algorithms expedite SLR for observational studies, thus allowing scientists to have more time to focus on the quality of data and the synthesis of evidence in observational studies. Aligning the living SLR concept, the system has the potential to update literature data and enable scientists to easily stay current with the literature related to observational studies prospectively and continuously.

## Introduction

Systematic literature reviews (SLRs) are widely recognized as a robust method to identify and summarize evidence from published sources [1]. However, conducting an SLR can be a complex, time-consuming, labor-intensive, and expensive task, depending on the breadth of the topic, level of granularity, or resolution of the review needed [2,3]. One recent study estimated the time and cost required to conduct an SLR can be as high as 1.72 person-years of scientist effort and approximately $140,000 per review [4]. Because SLRs are so resource intensive, it is difficult to stay up to date, and once an SLR is complete and new literature is published, the SLR may become incomplete and obsolete as time goes by.

Natural language processing (NLP) refers to artificial intelligence (AI) technologies that can extract structured information from textual documents such as medical charts, lab results, and many other types of unstructured text. NLP has significantly advanced a variety of biomedical applications in recent years. There is considerable community interest in using AI such as machine learning (ML) and NLP to improve automation in aspects of literature reviews [2,5-7]. For example, Thomas et al used NLP to identify randomized controlled trials for Cochrane reviews, and Wallace et al developed methods to extract sentences from literature related to clinical trial reports. There are also some SLR management software, such as Raynan.ai [8], which leverages NLP to expedite certain SLR steps, including article screening.

Despite these existing efforts, there is a lack of systematic and integrated NLP solutions for SLR to cover its full aspects, preventing the wide adoption of such tools in SLR projects.

Thus, in this study, we evaluated an intelligent SLR system (hereinafter referred to as ISLR) for observational SLR tasks. The use of NLP improves efficiency, while the human-in-the-loop approach improves accuracy and reduces errors. The system uses cutting-edge NLP tools that employ ML and deep learning (DL) approaches to expedite the time-consuming processes involved in an SLR by making a series of learned recommendations to the end user. The purpose of this study is to evaluate an AI tool that accelerates and streamlines the SLR process and to demonstrate the validity of this tool in 3 use cases.

## Methods

### Workflow and System Architecture

**Figure 1.**
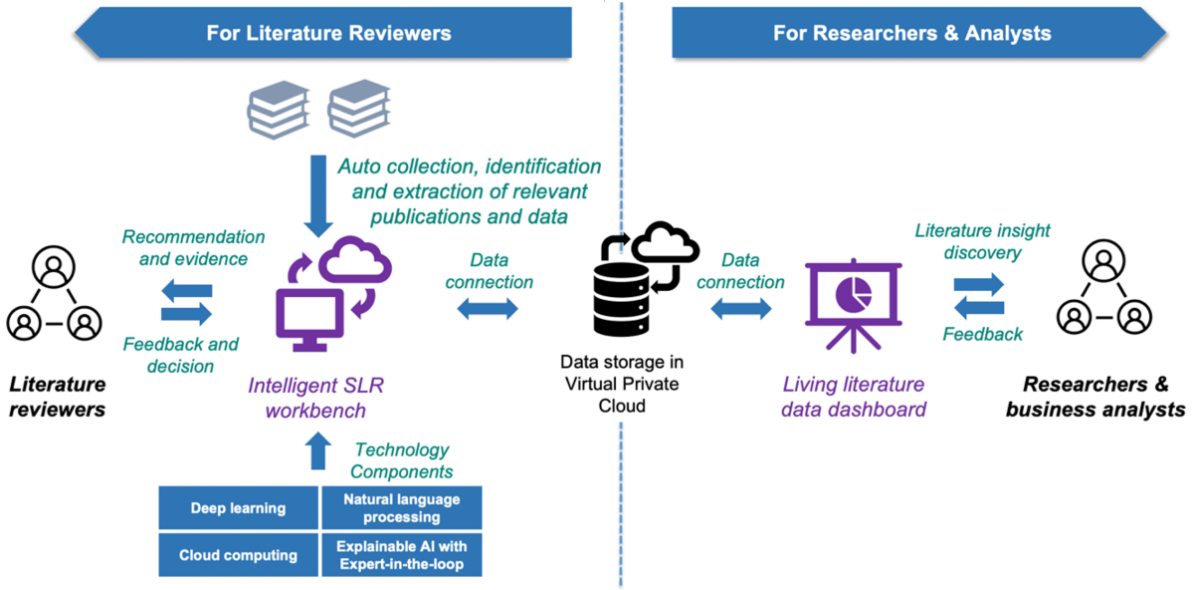
Overall data flow architecture of ISLR demonstrating the 2 major views. AI: artificial intelligence; ISLR: intelligent systematic literature review; SLR: systematic literature review.

ISLR has 2 major views that target 2 types of users in the observational studies in an SLR lifecycle: (1) an intelligent SLR workbench for literature reviewers who conduct routine literature reviews, and (2) a living literature data dashboard for researchers and analysts who focus on analyzing SLR data and keep up to date on new evidence. Figure 1 shows the overview architecture, including the 2 major views and data flow of the SLR system. ISLR integrates AI technologies and an SLR workflow management system to support literature collection, screening, and data extraction. The living literature dashboard continuously searches and updates the SLR, allowing users to interactively navigate the updated literature and develop new insights.

**Figure 2.**
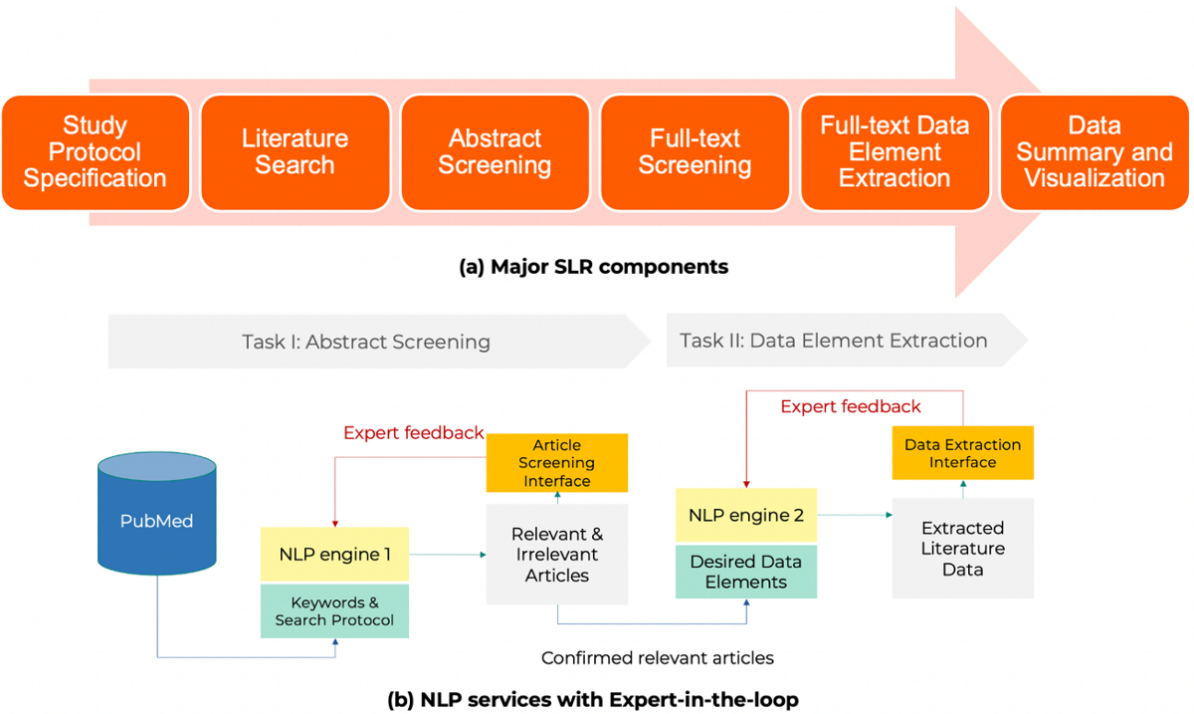
ISLR workflow and embedded NLP engines. ISLR: intelligent systematic literature review; NLP: natural language processing.

Reliable NLP systems depend heavily on the development of a reasonable workflow, user interfaces, and high-performance NLP algorithms. To develop the system and define the system workflow and user interfaces, we collaborated with end users who are experts in SLR using an iterative approach that employed industry-standard agile methodology. The team identified 6 major functional areas that were essential for the application: (1) protocol specification assistance, (2) literature search and indexing, (3) abstract screening with NLP assistance, (4) support for full-text searching, uploading, and screening, (5) full-text data element extraction using NLP assistance to identify and extract relevant data elements from full-text and embedded tables, and (6) literature data visualization to enable users to assess the SLR results and perform data discovery. Figure 2 shows the system workflow and the embedded NLP services to expedite two of the most time-consuming steps, which are article screening and data element extraction.

### Development and Validation of NLP Algorithms

**Figure 3.**
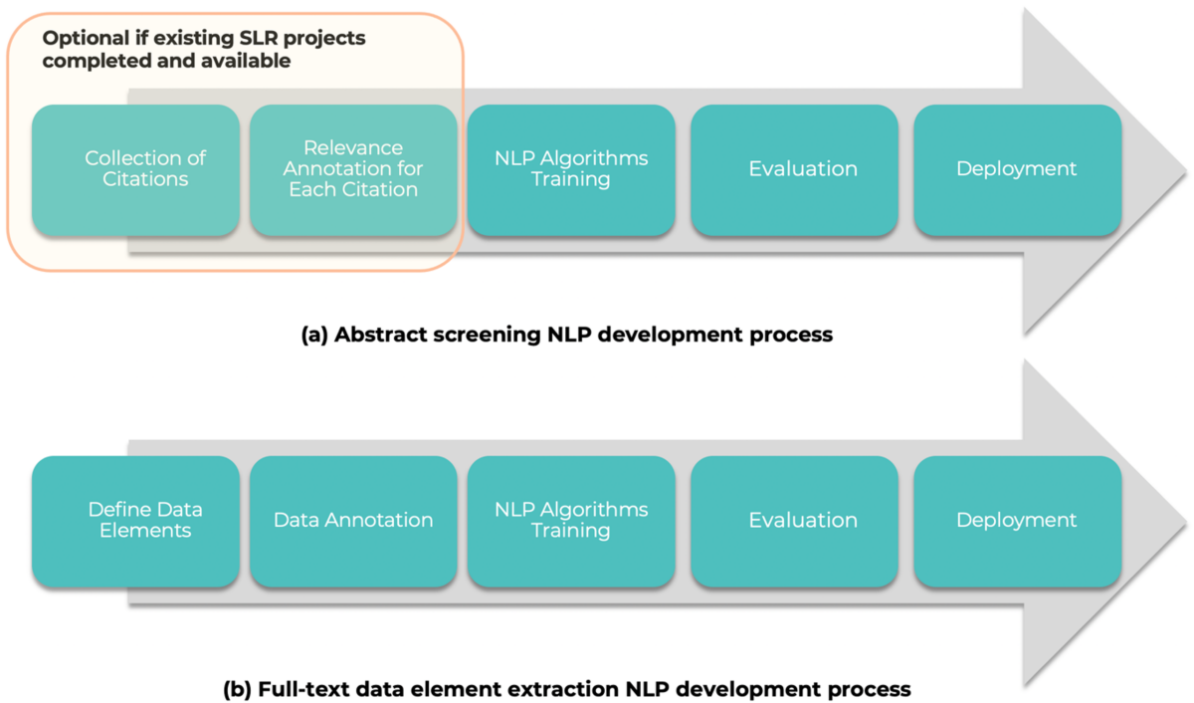
SLR NLP algorithm development steps. NLP: natural language processing; SLR: systematic literature review.

As mentioned earlier, 2 sets of NLP algorithms are required for a specific SLR project, including abstract screening and full-text data element extraction. Figure 3 outlines the NLP algorithm development process for these 2 steps separately. For abstract screening, the first step is to annotate and build a corpus that includes the abstract text, citation metadata, and inclusion/exclusion status. Once the corpus is prepared, NLP algorithm training, evaluation, and selection can be performed, and the best-performing algorithms will be chosen for deployment.

Similar to abstract screening, the NLP algorithm for the full-text data element extraction also requires a complete NLP development lifecycle. Unlike abstract screening, where labeled corpora may be available from previous SLR projects, data annotation is required to curate a labeled data set for training and evaluating NLP algorithms. The best-performing algorithms will be selected for deployment after evaluation. The following figure describes details on NLP algorithm development and validation process for SLR projects.

Three previously completed SLRs were used to guide and validate NLP development. These 3 projects included: (1) the prevalence of human papillomavirus (HPV) detected in head and neck squamous cell carcinomas (referred to as *HPV Prevalence*); (2) the epidemiology of the pneumococcal disease (referred to as *Pneumococcal Epidemiology*), and (3) the economic burden of pneumococcal disease (referred to as *Pneumococcal Economic Burden*). The inclusion and exclusion criteria for these 3 SLRs can be found in Table S1 in Multimedia Appendix 1.

#### Developing the Abstract Screening Corpora

Abstract screening was treated as a binary document classification task, ie, inclusion or exclusion of the article based on the abstract. Consequently, it was necessary to select and train NLP models for the task that demonstrated adequate performance and that had a reasonable computational time. The annotated screening literature sets from the 3 previous SLRs were used as the gold standard to train and validate models, including 1697, 207, and 421 articles for *HPV Epidemiology*, *Pneumococcal Epidemiology,* and *Pneumococcal Economic Burden*, respectively. The corpora contained citation metadata, including title, authors, Medical Subject Heading terms [9], and the text of the corresponding abstracts.

#### Developing the Full-Text Data Element Extraction Corpora

We selected 190, 25, and 24 full-text articles for *HPV Prevalence*, *Pneumococcal Epidemiology,* and *Pneumococcal Economic Burden* for annotation, respectively. Based on the key outcome variables defined in the 3 SLRs, we annotated 12 types of data elements, covering information related to general observational studies, such as *Study Population,* to disease-specific information such as *HPV Lab Technique* and *Pneumococcal Disease Type*.

#### Abstract Screening NLP Algorithms

For abstract screening, the NLP model classifies each article for its relevance based on its title, abstract, and other citation meta data. To build the abstract screening module, we evaluated 4 traditional ML-based document classification algorithms, XGBoost [10], support vector machines [11], logistic regression [12], and random forest [13] on the binary inclusion/exclusion classification task for abstract screening. The abstract screening corpora were used to evaluate NLP models by calculating standard metric of *precision (fraction of relevant instances among the retrieved instances, also called positive predictive value*), *recall (fraction of relevant instances that were retrieved, also called sensitivity*), *accuracy*, and *F1 scores* (the harmonic mean of precision and recall). The full features include title, abstract, authors, keywords, journal, Medical Subject Heading term, and publication types. We concatenated all features and extracted the term frequency-inverse document frequency vector as feature representation.

#### Data Element Extraction NLP Algorithms

To construct the module for data element extraction, we treated the problem of data element recognition and extraction as a named entity recognition (NER) problem, which aims to recognize the mentions of entities from the text [14]. We evaluated a series of NLP algorithms consisting of ML and DL algorithms to recognize and extract data elements from full text, including (1) conditional random fields (CRFs), a classic statistical sequence modeling algorithm that has been widely applied to NER tasks [15,16]; (2) long short-term memory (LSTM), a variation of recurrent neural networks that has achieved remarkable success in NER tasks [17,18]; and (3) “Clinical BERT (Bidirectional Encoder Representations from Transformers)” [19], a novel transformer-based DL model. Standard metrics, including *precision*, *recall*, *accuracy*, and *F1 scores*, were calculated.

### Ethical Considerations

This is not applicable as this study is not human subjects research.

## Results

Here, we report the results of the construction of the annotation corpora and the results of the NLP algorithm for abstract screening and data element extraction, respectively.

### Abstract Screening Corpora Description

The *HPV Prevalence* corpus we constructed from the existing SLR project contained 1697 total citations, of which 538 were included, and 1159 were excluded due to study criteria. The constructed *Pneumococcal Epidemiology* contained 207 citations, of which 85 were included and 122 were excluded. The constructed *Pneumococcal Economic Burden* corpus contained 421 citations, of which 79 were included, and 342 were excluded.

### Abstract Screening NLP Evaluation Results

Extensive studies have shown the superiority of transformer-based DL models for many NLP tasks [20-23]. Based on our experiments, however, adding features to the pretrained language models did not significantly boost their performance. The performance comparison results for each task are shown in Table 1. XGBoost achieved the highest accuracy on *HPV Prevalence* and *Pneumococcal Economic Burden* tasks, while a support vector machine achieved the highest accuracy on *Pneumococcal Epidemiology* task. XGBoost was ultimately chosen for deployment due to its better generalizability.

**Table 1.** Comparison of article screening natural language processing model performance.

Task and algorithm		F1 score	Precision	Recall	Accuracy
***HPV Prevalence*** **(n=1697)**					
	XGBoost	0.808	0.769	0.851	0.888
	Support vector machine	0.727	0.781	0.681	0.859
	Logistics regression	0.684	0.897	0.553	0.859
	Random forest	0.523	0.944	0.362	0.818
***Pneumococcal Economic Burden*** **(n=421)**					
	XGBoost	0.750	0.857	0.667	0.907
	Support vector machine	0.533	0.667	0.444	0.667
	Logistics regression	0.333	0.667	0.222	0.831
	Random forest	0.429	0.600	0.333	0.814
***Pneumococcal Epidemiology*** **(n=207)**					
	XGBoost	0.667	0.533	0.889	0.619
	Support vector machine	0.667	0.667	0.667	0.861
	Logistics regression	0.429	0.600	0.333	0.619
	Random forest	0.615	1.000	0.444	0.762

### Full-Text Data Element Extraction Corpora Description

The human annotators annotated 190, 25, and 24 full-text articles for the *HPV Prevalence*, *Pneumococcal Epidemiology*, and *Pneumococcal Economic Burden* tasks, respectively. Among these full-text articles, 4498, 579, and 252 entity mentions were annotated for 3 projects, respectively. However, the distribution of annotated entities is highly imbalanced. For example, data elements like *HPV Lab Technique* and *HPV Sample Type* were very prevalent, but data elements like *Maximum/Minimum Age in Study Cohort* were rarely annotated in the corpora.

### Results of the Full-Text Screening and Data Element Extraction NLP Methods

Tables2  3 show the comparison of NLP performance among CRFs, LSTM, and BERT on the 3 corpora. For each of the 3 corpora used to train the NLP models, LSTM demonstrated superiority over the conventional ML algorithm (ie, CRF) on entity recognition. Among DL models, we did not observe significant improvement in F1 scores by use of the BERT model. The BERT model achieved similar or worse performance on most data elements. The performance across different tasks varies, primarily due to the availability of annotated data. For example, on average, models’ performance on *HPV Prevalence* is higher than *Pneumococcal Epidemiology* and *Pneumococcal Economic Burden*, as *HPV Prevalence* has the largest annotated data. Due to the highly imbalanced distribution of annotated entities, we observe a variation in performance across different data elements for the same model. For example, in the *Pneumococcal Epidemiology* task, the LSTM model has achieved 0.412 in the identification of the *Study Cohort* and 0.768 in the identification of the *Pneumococcal Disease Type*.

**Table 2.** Overall performance comparison for the named entity recognition task in the 3 natural language processing training corpora. Scores averaged across all 12 extracted data elements. Measured in lenient F1 score.

Measure	*HPV Prevalence*			*Pneumococcal Epidemiology*			*Pneumococcal Economic Burden*		
	CRF^a^	LSTM^b^	Clinical BERT^c^	CRF	LSTM	Clinical BERT	CRF	LSTM	Clinical BERT
Microaverage (global average that uses the total number of true positives, false positives, and false negatives)	0.856	0.890	0.782	0.571	0.646	0.444	0.609	0.615	0.478
Macroaverage score (arithmetic mean of all the per-entity type scores)	0.522	0.674	0.685	0.270	0.295	0.227	0.216	0.238	0.231

a CRF: conditional random field.

b LSTM: long short-term memory.

c BERT: Bidirectional Encoder Representations from Transformers.

**Table 3.** Performance comparison for the named entity recognition task on selected data elements. Measured in lenient F1 score.

Measure	*HPV Prevalence*			*Pneumococcal Epidemiology*			*Pneumococcal Economic Burden*		
	CRF^a^	LSTM^b^	Clinical BERT^c^	CRF	LSTM	Clinical BERT	CRF	LSTM	Clinical BERT
*Study Cohort*	0.482	0.695	0.727	—^d^	0.412	0.278	—	—	—
*Study Location*	0.434	0.520	0.574	0.514	0.508	0.546	0.586	0.484	0.497
*Study Type*	0.733	0.760	0.753	0.364	0.525	—	—	0.328	0.299
*Pneumococcal Disease Type*	—	—	—	0.725	0.768	0.526	0.644	0.715	0.523
*Incidence or Prevalence*	0.986	0.983	0.924	—	—	—	—	—	—
*Study Time*	0.714	0.888	0.930	0.222	0.636	0.328	—	—	—

a CRF: conditional random field.

b LSTM: long short-term memory.

c BERT: Bidirectional Encoder Representations from Transformers.

d Not applicable.

### Final NLP Algorithm Selection

NLP algorithms were needed for the 2 tasks, abstract screening, and data element extraction, in the ISLR system. The abstract screening was treated as a classification task. Based on our experimental results, XGBoost was selected for this task due to good performance on our document classification experiments and less computational complexity than DL-based models. For the data element extraction task, LSTM was selected over CRF and BERT for the same reasons.

### ISLR System Components

#### Study Protocol Specification

Study protocol specification is one of the first steps in an SLR project. Users can upload a PDF document to the system that describes the SLR study protocol for reference. The SLR system has a default list of data elements with their descriptions and answer types (eg, free text, multiple choice, and checkbox), which will be extracted from full-text PDFs of articles. The system also allows users to create and modify the list. At the end of the project, all the extracted data elements can be exported in a structured format.

#### Literature Search

The ISLR system is integrated with the PubMed E-utilities application programming interface, which enables users to perform direct searches on PubMed. Citation metadata such as abstracts, titles, journals, and authors can be retrieved from PubMed and indexed in the system for further screening and data element extraction. Additionally, the system provides an option for users to retrieve this citation metadata by uploading a list of individual PubMed IDs.

#### Abstract Screening

**Figure 4.**
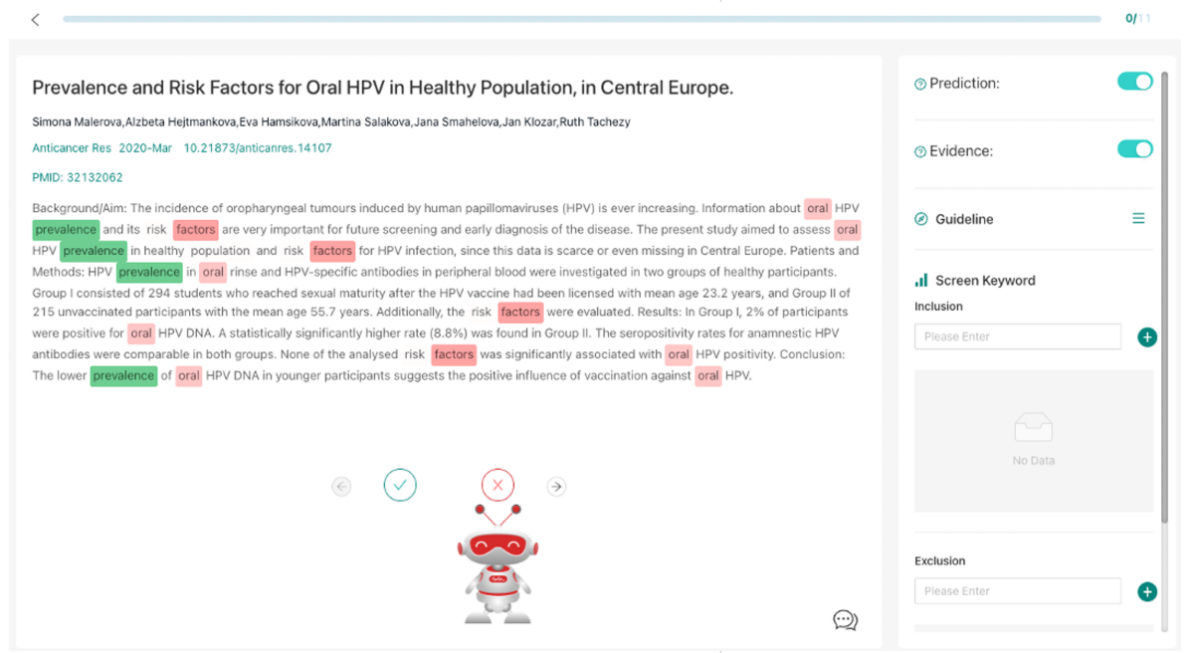
Abstract screening interface. Terms that support inclusion in the finalized cohort of relevant articles are shown in green, while terms that detract from inclusion are shown in red. The scale of the colors shows how significantly one term can impact prediction decisions (eg, darker color indicates higher impact).

The purpose of abstract screening is to review collected articles’ relevance based on their title, abstract, and other relevant metadata, such as journal names, article types, and keywords. The relevant articles will be included for the following full-text screening and data element extraction steps. NLP services are provided at this step to make recommendations on whether a particular article should be included for full-text review. The supporting information (eg, salient words that are impactful to inclusion and exclusion) for the NLP recommendation will also be shown to provide explainable evidence. Human experts can further review the predictions for each article and decide on abstract screening status (keep or exclude). Figure 4 shows the abstract screening interface demonstrating prediction results and relevant terms discovered by the NLP algorithms.

#### Full-Text Searching, Uploading, and Screening

This step aims to identify full-text PDF documents for each included article and further screen their relevance based on the SLR study protocol. Only the articles that are deemed relevant after this stage will be included in the final full-text data element extraction step. The process of locating full-text PDF documents for each article can be time-consuming. The ISLR system integrates with PubMed Central to automatically find and collect full-text PDFs if they are publicly available. However, for articles whose full-text PDFs are not publicly available, users need to manually locate the articles through publishers and upload the corresponding PDFs to the system through the provided user interface.

#### Full-Text Data Element Extraction

**Figure 5.**
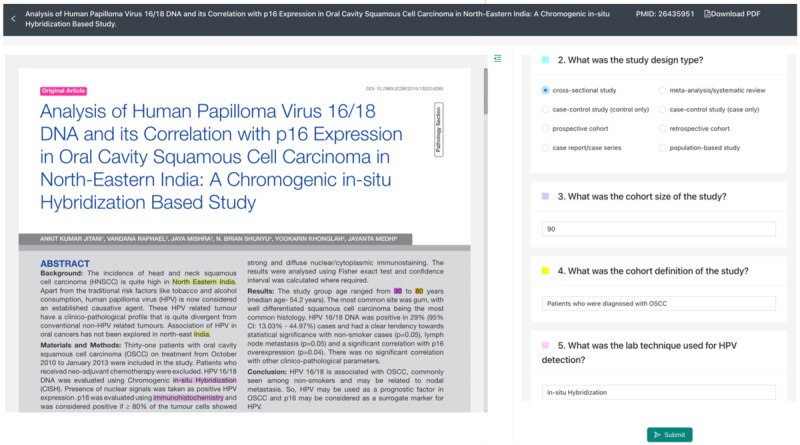
Full-text data element extraction user interface. Data elements from the article extracted by the NLP algorithms are color-coded and highlighted in the PDF. Highlight colors in the PDF text are linked to the data elements as shown in the right-hand frame. For the data element list on the right side, all the extracted data elements can pop up as candidates for the users to choose from. NLP: natural language processing.

Extracting full-text data elements is a time-consuming process in SLR projects. It requires reviewing the full-text article and extracting multiple relevant pieces of information defined in the study protocol. These data elements are often found in various sections of an article, including tables. The ISLR system uses Amazon Textract [24] for optical character recognition to extract text and tables from PDF files, followed by NLP services to further extract information from both text and tables. The NLP services can recommend potential answers for each data element, and human experts can review, select, and modify the extracted information. Figure 5 shows a screenshot of the user interface for this step.

#### Data Summary and Visualization

**Figure 6.**
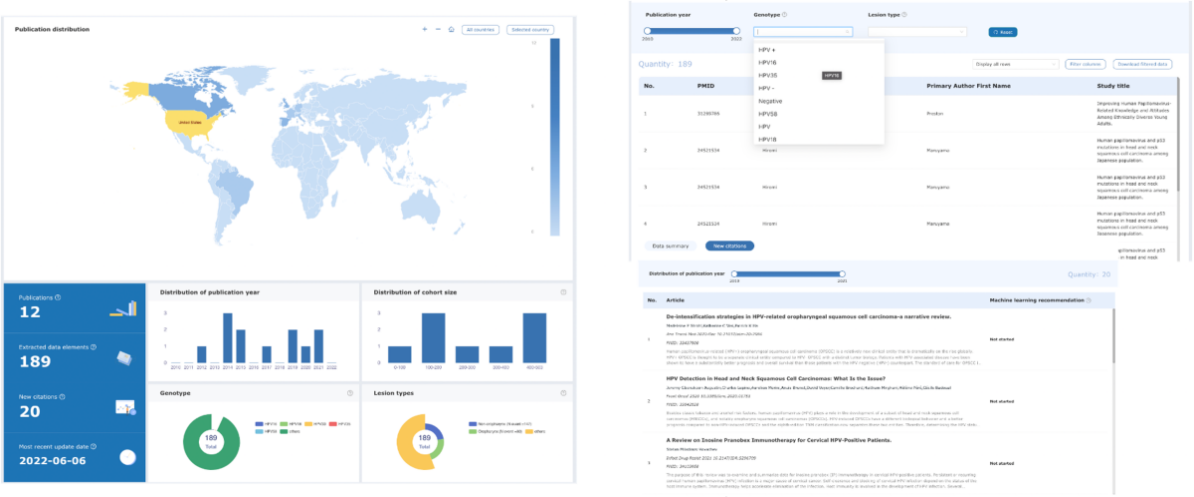
Interactive visualization of existing SLR data, lists of relevant publications, and data exportation control. SLR: systematic literature review.

The ISLR system offers interactive dashboards to end users, such as researchers, for exploring the SLR results and data. These dashboards allow users to apply data filters, such as study location and cohort size, to refine their search results. For each data element extracted from full-text articles, users can click on the element to navigate to the corresponding article, ensuring traceability and appropriate references to source documents in the SLR project. Additionally, the dashboards recommend recent relevant articles and suggest articles that may require full-text screening. Figure 6 displays the major functions and screenshots of the dashboard.

## Discussion

### Principal Findings

As described in the introduction, conducting an SLR is complex and expensive. There is also a rapid growth of the available number of publications and other data, such as clinical trial reports used in the article search and screening processes, with an average annual growth rate for the life sciences of around 5% [25]. Consequently, there is considerable community interest in applying various types of automation, including AI, DL, and NLP, to the multiple tasks required for producing an accurate SLR [2,5-7].

An important consideration for using the results of an SLR is how often the SLR is updated and hence how timely and complete these data are with respect to the real-world evidence. “Living” ISLR system addresses the difficulty of updating an SLR by providing an automated workflow including review tools to detect when new data are available and to trigger at least a semi-automated update process for the expedited review. The system is also expandable to cover additional data elements of interest by updating existing NLP pipelines.

The major accomplishments of this ISLR system include improving the time, efficiency, cost, completeness of evidence, and error avoidance through techniques to assist researchers with decision-making (so-called human-in-the-loop). The ISLR system is aligned with the living SLR concept, as it supports a rapid update of existing literature data. Additionally, since the classification and data element extraction tasks are maintained by the system, results can be used for retraining the classification and NLP algorithms on a routine basis. Consequently, the performance of the system should improve over time.

The focus of this work was to evaluate an intelligent system that includes all major steps of an SLR with humans in the loop. The corpora evaluated in this study mostly focus on health economics and outcomes research in specific therapeutical areas. The generalizability of the learning algorithms to another domain will benefit from further formal examination. Since we have not yet conducted a time analysis of an SLR study conducted both manually and with this tool, we are unable to precisely quantify the time savings from the ISLR system. In addition, our NLP technologies limit to the extraction of relevant information directly from the text but are not able to conduct reasoning with long context to support complex data element extraction, such as GRADE (Grading of Recommendations, Assessment, Development, and Evaluation) or RoB2 (Risk of Bias 2). The recent advances in large language models, such as generative pretrained transformer 4, bring NLP technologies expert-level performance on various professional and academic benchmarks. Given its high performance, generalizability, and reasoning capacity, it would be interesting to further assess the efficacy and accuracy of large language models in various SLR tasks and complex data element extraction.

As an early and innovative attempt to automate SLR lifestyle through NLP technologies, ISLR does not fully support PRISMA (Preferred Reporting Items for Systematic Reviews and Meta-Analyses) reporting yet. We plan to continuously iterate ISLR to cover the PRISMA checklist and report generation in the future. In addition, we have not yet conducted formal usability studies of the user interface, although agile methods involving iterative refinement of the interface through input from domain experts in SLR were employed throughout the software development process.

### Conclusions

Our ISLR system is a user-centered, end-to-end intelligent solution to automate and accelerate the SLR process and supports “living” SLRs with humans in the loop. The system integrates cutting-edge ML- and DL-based NLP algorithms to make recommendations on article screening and data element extraction, which allow the system to prospectively and continuously update relevant literature in a timely fashion. This allows scientists to have more time to focus on the quality of data and the synthesis of evidence and to stay current with literature related to observational studies.

## Supplementary material

10.2196/54653 Multimedia Appendix 1 Inclusion and exclusion criteria for 3 systematic literature review projects.

## Abbreviations

AI: artificial intelligence
BERT: bidirectional encoder representations from transformers
CRF: conditional random field
DL: deep learning
GRADE: Grading of Recommendations, Assessment, Development, and Evaluation
HPV: human papillomavirus
ISLR: intelligent systematic literature review
LSTM: long short-term memory
ML: machine learning
NER: named entity recognition
NLP: natural language processing
PRISMA: Preferred Reporting Items for Systematic Reviews and Meta-Analyses
RoB2: Risk of Bias 2
SLR: systematic literature review
