# Supplementary material for: Accelerating Evidence Synthesis in Observational Studies: Development of a Living Natural Language Processing–Assisted Intelligent Systematic Literature Review System
Source: JMIR Med Inform. 2024 Oct 23;12:e54653. doi: 10.2196/54653 (PMC11523763; doi:10.2196/54653)
Supplement: Multimedia Appendix 1 [file medinform-v12-e54653-s001.docx]

**Table S1. Inclusion & exclusion criteria for three SLR projects**

|  | HPV Prevalence | Pneumococcal Epidemiology | Pneumococcal Economic Burden |
| --- | --- | --- | --- |
| Purpose of SLR | To identify the available peer-reviewed evidence on the prevalence of HPV detected in head and neck squamous cell carcinomas (HNSCCs). | To understand the epidemiology of the pneumococcal disease (both invasive and noninvasive, caused by Streptococcus pneumonia) in children and adults in 13 countries of interests | To understand the economic burden of the pneumococcal disease (both invasive and noninvasive, caused by Streptococcus pneumonia) in children and adults in 13 countries of interests |
| Populations | adults (age >= 13) with histologically confirmed invasive HNSCCs (oral cavity, oropharynx, larynx, hypopharynx) | Populations with Invasive and noninvasive pneumococcal disease  (Excluding children < 5 years and adults > 18 years) | Populations with Invasive and noninvasive pneumococcal disease  (Excluding children < 5 years and adults > 18 years) |
| Primary Outcomes | overall HPV prevalence and/or type distribution (types 16 and 18 and at least one of the new Gardasil 9 vaccine types [i.e., types 31, 33, 45, 52 or 58]) | Epidemiology data (incidence, prevalence, etc.) of invasive or noninvasive pneumococcal disease | Direct resource use or costs by health state; Indirect or other resource use or costs of interest; quality-of-life data |
| Time of Publications | Publication date from 2015 to 2020 | Publication date from 2012 to 2017 | Publication date from 2012 to 2017 |
